# The clinical and genetic characteristics of permanent neonatal diabetes (PNDM) in the state of Qatar

**DOI:** 10.1002/mgg3.753

**Published:** 2019-08-23

**Authors:** Sara Al‐Khawaga, Idris Mohammed, Saras Saraswathi, Basma Haris, Reem Hasnah, Amira Saeed, Hakeem Almabrazi, Najeeb Syed, Puthen Jithesh, Ahmed El Awwa, Amal Khalifa, Fawziya AlKhalaf, Goran Petrovski, Essam M. Abdelalim, Khalid Hussain

**Affiliations:** ^1^ College of Health & Life Sciences Hamad Bin Khalifa University, Qatar Foundation Doha Qatar; ^2^ Division of Endocrinology, Department of Pediatric Medicine Sidra Medicine Doha Qatar; ^3^ Diabetes Research Center Qatar Biomedical Research Institute, Hamad Bin Khalifa University, Qatar Foundation Doha Qatar; ^4^ Biomedical Informatics Division Sidra Medicine Doha Qatar; ^5^ Faculty of medicine Alexandria University Alexandria Egypt

**Keywords:** Fanconi–Bickel Syndrome (FBS), *GCK*, *HNF1B*, *INS*, pancreatic agenesis, Permanent neonatal diabetes (PNDM), *PTF1A*, Whole Genome Sequencing (WGS), Wolcott–Rallison Syndrome (WRS)

## Abstract

**Background:**

Neonatal diabetes mellitus (NDM) is a rare condition that occurs within the first six months of life. Permanent NDM (PNDM) is caused by mutations in specific genes that are known for their expression at early and/or late stages of pancreatic beta‐ cell development, and are either involved in beta‐cell survival, insulin processing, regulation, and release. The native population in Qatar continues to practice consanguineous marriages that lead to a high level of homozygosity. To our knowledge, there is no previous report on the genomics of NDM among the Qatari population. The aims of the current study are to identify patients with NDM diagnosed between 2001 and 2016, and examine their clinical and genetic characteristics.

**Methods:**

To calculate the incidence of PNDM, all patients with PNDM diagnosed between 2001 and 2016 were compared to the total number of live births over the 16‐year‐period. Whole Genome Sequencing (WGS) was used to investigate the genetic etiology in the PNDM cohort.

**Results:**

PNDM was diagnosed in nine (*n* = 9) patients with an estimated incidence rate of 1:22,938 live births among the indigenous Qatari. Seven different mutations in six genes (*PTF1A, GCK, SLC2A2, EIF2AK3, INS,* and* HNF1B*) were identified. In the majority of cases, the genetic etiology was part of a previously identified autosomal recessive disorder. Two novel de novo mutations were identified in *INS* and *HNF1B*.

**Conclusion:**

Qatar has the second highest reported incidence of PNDM worldwide. A majority of PNDM cases present as rare familial autosomal recessive disorders. Pancreas associated transcription factor 1a (*PTF1A)* enhancer deletions are the most common cause of PNDM in Qatar, with only a few previous cases reported in the literature.

## 1 INTRODUCTION

Neonatal diabetes mellitus (NDM) or “early‐onset” diabetes is a rare form of diabetes characterized by hyperglycemia that presents during the first six months of life. Intrauterine growth retardation (IUGR), failure to thrive, and low C‐peptide levels are common clinical and biochemical features in NDM (Aguilar‐Bryan & Bryan, 2008; von Muhlendahl & Herkenhoff, 1995). NDM is estimated as one case per 300,000–500,000 live births (Polak & Cave, 2007; Polak & Shield, 2004). Two main types, transient NDM (TNDM) and permanent NDM (PNDM), have been described based on the duration of insulin dependency. TNDM represents 50%–60% of cases and usually resolves before 18 months of age (von Muhlendahl & Herkenhoff, 1995). PNDM is less common than TNDM and by definition, does not go into remission. NDM is a monogenic disorder that occurs due to mutations in genes that play an important role in pancreatic development, beta‐cell survival, insulin processing, regulation, and release. Currently mutations in more than 20 different genes have been identified in patients with PNDM (Flanagan et al., 2014; Rubio‐Cabezas & Ellard, 2013).

Heterozygous mutations in the potassium voltage‐gated channel subfamily J member 11 (*KCNJ11)* and ATP binding cassette subfamily C member 8 (*ABCC8)* impact the role of the ATP‐sensitive potassium channel (K_ATP_) in the beta‐cell membrane, and are the most common cause of PNDM in the Western world (Flanagan, Edghill, Gloyn, Ellard, & Hattersley, 2006; Russo et al., 2011). These mutations account for 31% and 10% of all PNDM cases, respectively (Babenko et al., 2006; Gloyn et al., 2004). Most patients with *KCNJ11* and *ABCC8* mutations have isolated diabetes, and their treatment can be switched from subcutaneous insulin injections to treatment with oral sulfonylureas (SU) (Bowman et al., 2018; Pearson et al., 2006).

The frequency of PNDM is estimated to be around one in 200,000 live births in countries with low rate of consanguineous marriages (Barbetti & D'Annunzio, 2018; Iafusco et al., 2012; Polak & Cave, 2007; Stanik et al., 2007) while the highest incidence of PNDM has been reported in the Northwest region of Saudi Arabia (Habeb et al., 2012). In consanguineous families with high level of homozygosity, PNDM is commonly associated with syndromic forms of DM, with recessive eukaryotic translation initiation factor 2‐alpha kinase 3 (*EIF2AK3*) mutations causing Wolcott–Rallison Syndrome (WRS) (OMIM 226980) being the most frequent cause (Habeb et al., 2012; Rubio‐Cabezas & Ellard, 2013).

The unique population in the State of Qatar of over 2.6 million people has primarily arrived from the Middle East and North Africa (MENA) and South Asia. Around 15% are indigenous Qataris of Arabian Peninsula ancestries, who have also immigrated to the State in the past decades (Fakhro et al., 2016). The native Qataris represent a population with a combination of Bedouin (Q1), South Asian (Q2) and African (Q3) descent, contributing to the observed diversity at the genome level (Fakhro et al., 2016; Hunter‐Zinck et al., 2010; Rodriguez‐Flores et al., 2014). Specifically, the Bedouin subpopulation continues to practice within‐tribal marriages leading to high levels of homozygosity compared with other populations (Hunter‐Zinck et al., 2010; Rodriguez‐Flores et al., 2016). The clinical and genetic analysis of PNDM along with rare genetic disorders from The State of Qatar has not been studied before. Guidelines for the management of complex syndromes also remain far from clear and thus encourage ongoing studies to elucidate novel genetic and clinical characteristics to help in an improved diagnosis, prevention strategies, clinical management and aid in a proper prognostic evaluation.

Human genome reference used for variant detection that perfectly resembles the ancestry of the genome(s) being aligned is anticipated to decrease mismatches during alignment ultimately resulting in more precise genotypes (Dewey et al., 2011). Since allele frequency is population‐dependent, relying solely on the standard reference genome (GRCh37) or allele frequency in ethnically mismatched populations may result in inadequate evaluations of the pathogenicity of a specific allele in a poorly genotyped population such as The Arabian Peninsula (Dewey et al., 2011; Richards et al., 2015). Therefore, The State of Qatar, known to have a high prevalence of homozygous disorders, could benefit significantly from a precise major allele reference genome and frequency database of ethnically matched controls (Lucassen & Houlston, 2014). To produce the first NDM reference genome version designed for the Arabian Peninsula population, we have sequenced genomes from Qatari PNDM patients. In this study, we aim to understand and define the clinical characteristics, explore the genotype and phenotype of patients with PNDM diagnosed in the State of Qatar, and estimate the incidence of PNDM during the period 2001–2016. By integrating genome sequencing data with functional annotation from ENCODE (Consortium, 2012) and the Epigenome Roadmap (Bernstein et al., 2010), we were able to discover causal alleles in the PNDM cohort. Interestingly, knowledge gained from genome sequencing data combined with discovery of novel mutations in regulatory noncoding regions continues to provide new perspectives into monogenic disorders in highly consanguineous populations (Weedon et al., 2014). In this study, we have identified several autosomal recessive disorders associated with PNDM, including isolated pancreatic agenesis (OMIM 615935) due to pancreas associated transcription factor 1a (*PTF1A)* mutation, Fanconi–Bickel syndrome (FBS) due to solute carrier family 2 member 2 (*SLC2A2)* mutation (OMIM 227810), Wollcot–Ralison Syndrome (WRS) due to eukaryotic translation initiation factor 2 alpha kinase 3 (*EIF2AK3)* mutation (OMIM 226980), and homozygous recessive glucokinase (*GCK,* OMIM 138079) and insulin (*INS,* OMIM 176730) mutations. Several de novo mutations in*INS* and HNF1 homeobox B (*HNF1B,* OMIM 137920) are also described. The clinical spectrum and associated genetic characteristics governing the cohort's PNDM is also discussed.

## 2 METHODS

### 2.1 Ethical compliance

This study was approved by the Institutional Review Board (IRB) for the protection of human subjects in Sidra Medicine, Qatar.

### 2.2 Study participants

This study was performed in Sidra Medicine, where children diagnosed with DM were referred. Sidra Medicine is the only National Referral Center for all children diagnosed with DM; therefore, we have captured all the NDM cases. Children who were diagnosed with DM within six months of life were labeled as NDM. Clinical details related to their diabetes onset, birth weight, gestational age, family histories, and prognosis were collected. The genomic DNA of the samples collected from patient and family members was isolated from peripheral blood samples. The extraction and purification were conducted according to the manufacturer's protocol (QIAamp DNA Blood Maxi Kit, Qiagen, Cat. No.: 51194). Pedigrees were constructed and drawn using the genetic data management system, Progeny Clinical – Version N from Progeny Genetics (Progeny Genetics LLC, Delray Beach, FL, www.progenygenetics.com).

### 2.3 Epidemiological analysis: Incidence of PNDM

Dividing the total number of patients diagnosed with PNDM between 2001 and 2016 by the total number of live births in the State of Qatar over the 16‐year‐period was used to calculate the incidence of PNDM. The Ministry of Public Health in Qatar has confirmed the annual live birth rate from 2001 to 2016.

### 2.4 Genetic analysis

#### 2.4.1 Genome sequencing and data filtering

DNA was extracted from the blood specimen of 28 individuals (including trios; patients and parents) in Sidra Medicine, Qatar. Whole Exome Sequencing (WES) was initially used on patient samples. The generated whole exome sequence data from patients’ genomic DNA sample and additional familial specimens were reannotated and reanalyzed in comparison with the published human genome build GRCh37/UCSC hg19, and analyzed for sequence variants. Mean depth of coverage was 112×, which refers to the mean number of sequence reads obtained across the whole exome. The quality threshold, which is covered by at least 10 sequence reads (10× coverage), allowed for high‐quality exome variant base calling, annotation, and evaluation. Whole Genome Sequencing (WGS) was performed on patient and related family specimen when WES analysis was inconclusive. As a first step, the extracted DNA was used for WGS on Illumina HiSeqX platform using a 150‐base paired‐end single‐index‐read format. The read depth used for WGS ranged from 30–35× coverage. This method yielded two FASTQ files that contain the nucleotide sequence reads and quality scores for each sample.

FASTQ files, that contain the nucleotide sequence reads and quality scores, were generated for each sample. Burrows–Wheeler Aligner (BWA‐MEM, version 0.7.8) was used to map the sequence reads, with default parameters, for each individual to the NCBI human reference genome GRCh37/hg19. This resulted in aligned reads in sequence alignment map format (SAM), which were then converted to sorted, indexed binary alignment map (BAM) format using SAMTools, version 1.7. Picard tools (version 2.6.0) were used to mark duplicated reads. GATK software tools (version 3.4; http://www.broadinstitute.org/) were used to improve the BWA‐MEM alignments and genotype calling using the GATK recommended best practices, which is summarized as follows. The BAM files generated by the BWA‐MEM aligner were realigned with the GATK base IndelRealigner. The GATK base quality recalibration tool was used to recalibrate the base quality scores. Genotypes were called by the GATK Haplotypecaller, and the GATK VariantRecalibrator tool was used to score the variant calls. The learning algorithm was used to identify high‐quality SNPs and INDELs using the Variant Quality Score Recalibration (VQSR) procedure. Joint‐genotype across individuals within each family was performed using the GATK GenotypeGVCFs tool.

SNPEff (Geoffroy et al., 2015; van der Velde et al., 2017), an annotation tool that can predict the effects due to variations in the genetic code that might result in amino acid changes, was used to enhance the annotations in the VCF files. Additionally, vcfanno (Pedersen, Layer, & Quinlan, 2016) was used to annotate VCF file with extensive available data resources like the Genome Aggregation Database (gnomAD) (Lek et al., 2016), 1,000 genome (Clarke et al., 2017), Combined Annotation‐Dependent Depletion (CADD) (Rentzsch, Witten, Cooper, Shendure, & Kircher, 2018), and GERP (Paila, Chapman, Kirchner, & Quinlan, 2013). Each individual's family annotated VCF file was uploaded to the GEnome MINIng tool (GEMINI) (Paila et al., 2013) using vcf2db utilities for querying.

Quality control (QC) metrics were collected at different stages of the analyses, to ensure the accuracy of the data. FastQC (version 0.11.2) software was run on the raw data while combinations of SAM Tools (version 1.7) and Picard (version 2.6.0) packages were used to analyze the QC of the mapped reads. To evaluate the provided pedigree information, Peddy (Pedersen & Quinlan, 2017) was run independently for each family to infer the familial relationship for each reported families from the genotyped data. Integrative Genomics Viewer (IGV) was further used to validate the variants. Figure S1a illustrates the variant classification process.

#### 2.4.2 Structural variants analysis

Structural variants discovery for these individuals was performed using the DELLY (Rausch et al., 2012), which integrates short insert paired‐ends, long‐range mate‐pairs and split‐read alignments to delineate genomic rearrangements at single‐nucleotide resolution. The structural variation (SV) calling was done for each sample and SV sites were merged into one unified list. Finally, genotyping the merged SV site list across all samples was done to get a final VCF file. Samplot software was used to visualize the deleted regions upstream of PTF1A (https://github.com/ryanlayer/samplot).

#### 2.4.3 Sanger sequencing

To confirm the mutations in the patient and both parents, the extracted genomic DNA was used for Sanger sequencing (Estrada‐Rivadeneyra, 2017). For each gene, specific primers were designed. The complete list of primers is presented in Table S1. Applied Biosystems Sequencing Analysis Software v6.0 (Sequencing Analysis Software v6.0, Applied Biosystems, Cat No.: 4474950), and Applied Biosystems SeqScape Software v3.0 were used for Sanger sequencing analysis (SeqScape^TM^ Software v3.0, Applied Biosystems, Cat No.: 4474978).

## 3 RESULTS

### 3.1 Incidence of PNDM

The current study included nine patients from seven first‐degree consanguineous families that were born in the State of Qatar and diagnosed with PNDM between 2001 and 2016. The total number of PNDM patients (*n* = 9) accounts for approximately 12% of the diabetic children diagnosed before the age of 5 years in Qatar. Based on the country's total birth rate during the 16‐year‐period, we divided the incidence in the Qatari population into three main categories; the indigenous Qatari, non‐Qatari, and total Qatari population. Table S2 shows the total number of live births from 2001 to 2016 in the indigenous Qatari and nonindigenous Qatari population. The incidence of PNDM during the period 2001–2016 is 1:22,938 live births among the indigenous Qatari population. The calculated incidence per 1,000,000 births in the indigenous Qatari population is 43.6 (95% CI per 1,000,000 births is 14.2–101.7), 22.2 in the nonindigenous Qatari population (95% CI per 1,000,000 births is 6.0–56.7), and 30.5 in the total population living in the State of Qatar (95% CI per 1,000,000 births is 13.9–57.9). Figure S1b, provides a comparison of the incidence rate of PNDM in the State of Qatar compared to the other highest worldwide reported countries, the indigenous Qataris being the second highest

### 3.2 Phenotype and genome analysis

Genetic mutations were identified in the whole cohort (100%). The clinical and genetic characteristics identified are shown in Table 1 and Figure S1c. Seven different mutations in six different genes (*PTF1A, GCK, SLC2A2, EIF2AK3, INS,* and* HNF1B*) were identified, however, no patient had *KCNJ11* or *ABCC8* mutations. A majority of patients had a clear genetic etiology in which their PNDM was part of autosomal recessive syndromes or rare disorders due to abnormal early pancreatic development, including Wolcott–Rallison Syndrome (WRS), Fanconi–Bickel syndrome (FBS), and pancreatic agenesis due to *PTF1A* enhancer deletion, respectively.

**Table 1:** Clinical and genetic characteristics of the Qatari PNDM cohort

Case number	Clinical characteristics, biological, and genetic profile at time of diagnosis						Remarks
	Gestational age	Birth Weight (kg)	Age at diagnosis (Days)	Fasting blood glucose (mmol/L)	C‐peptide (ng/mL)	Gene mutation	
1^a^	Term	1.3	1	7.7	–	*PTF1A* (chromosome 10:23502416–23510031)	Pancreatic agenesis/atrophy, short stature^b^
2^a^	Term	1.0	1	13.5	–	*PTF1A* (chromosome 10:23502416–23510031)	Pancreatic agenesis/atrophy and short stature^b^
3^a^	Term	1.9	1	12.3	0.02	*PTF1A* (chromosome 10:23502416–23510031)	Pancreatic agenesis/atrophy^b^
4^a^	35 weeks	1.5	1	13.5	0.11	*GCK* (c.437T > C)	b
5^a^	Term	2.0	18	10.1	0.33	*SLC2A2* (c.901C > T)	Fanconi–Bickel Syndrome, renal tubular acidoisis, short stature^b^
6^a^	Term	2.7	120	9.1	0.15	*EIF2AK3* (c.1570_1573delGAAA)	Wolcott–Rallison Syndrome (WRS)^b^
7^a^	Term	1.6	26	33	0.29	Recessive *INS* (c.‐331C > G)	b
8^a^	Term	3.0	60	14.0	0.68	*de novo INS* (c.325T > A)	–
9^a^	Term	1.9	3	29.6	0.01	*HNF1B* (c.1099A > G)	Hypothyroidism, hepatosplenomegaly, electrolyte imbalance^b^

Reference range: C peptide 0.78 – 2.83 ng/mL; Fasting blood glucose – 3.3‐5.5 mmol/L.

a Autoimmune markers tested negative.

b First‐degree consanginous parents.

Birth weight (BW; Kg), age at diagnosis, glycemic status, C‐peptide level, and the clinical spectrum of all patients are displayed in Table 1. Out of the nine patients, six were males resulting in a a male to female ratio of 2:1. The mean BW was 1.9 kg and mean age at diagnosis was 3.6 weeks. The majority of the patients (89%) were born to first‐degree consanguineous parents. One patient died before testing (sibling of patient 5); who was diagnosed with WRS.

**Figure 1:**
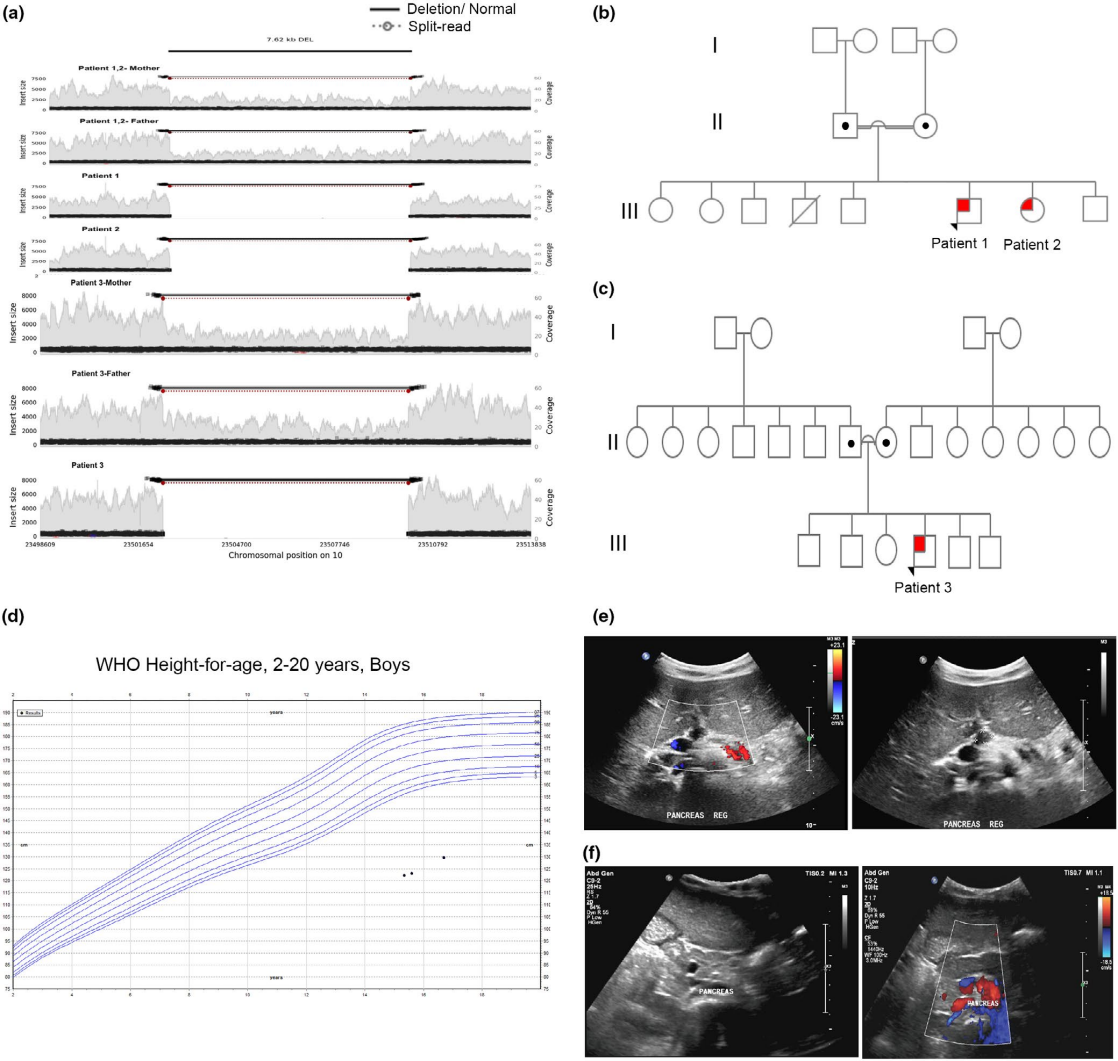
Patients with *PTF1A* distal enhancer deletion. (a) WGS analysis showing structural variant (7.62 kb deletion) in chromosome 10:23502416–23510031, located downstream of *PTF1A*. Pedigree chart of two unrelated consanguineous families, family 1 (b) and family 2 (c). Squares indicate male persons, and circles female persons. The arrow indicates the proband. The open symbols, dotted symbols, and solid symbols represent nonvariant, heterozygote, and homozygous for mutations, respectively. (d) WHO for the height of patient 1. Ultrasound (US) of the abdomen showing pancreatic atrophy (e, f) in patient 1 and 3, respectively. The white arrow points to the atrophied pancreas

Linkage and whole genome sequencing analysis was used to identify the mutations causing isolated pancreatic agenesis in two unrelated consanguineous Q1 families. Homozygosity mapping in three affected and four unaffected subjects from the two families revealed a single shared locus on chromosome 10 (Figure 1a) that included PTF1A (NM_178161)*.* Mutations in coding and promoter sequences of *PTF1A* were excluded. The previously reported sequence (chromosome 10:23502416–23510031) is located ~ 25 kb downstream of *PTF1A.*

The three patients (case No. 1–3) with *PTF1A* enhancer deletion and isolated pancreatic agenesis descend from two unrelated consanguineous families (Figure 1b,c). Case No. 1 is a 17‐year‐old Qatari (Q1) male who is the sixth child of first‐degree consanguineous parents, diagnosed with pancreatic agenesis during early neonatal period. Among other features are short stature (Figure 1d), microcephaly, global developmental delay, and seizure disorder attributed to cerebral palsy. Brain CT confirmed normal brain parenchyma, cerebral ventricles, and preserved basal cisterns. Ultrasound (US) of the abdomen showed that the pancreatic head appeared echogenic and atrophied (Figure 1e). Case No. 2 (sibling of case No. 1) is a 14‐year‐old girl; seventh child of first‐degree consanguineous parents diagnosed with PNDM and isolated pancreatic agenesis during early neonatal period.

Case No. 3 is a 5‐year‐old boy of Arabian Gulf origin. The patient is the fourth child of first‐degree consanguineous parents. The patient was diagnosed with PNDM in the neonatal intensive care unit (NICU), when he was just a day old. Later, the patient was diagnosed with pancreatic insufficiency with a stool elastase level of <15 ug/gm indicating severe exocrine pancreatic insufficiency, confirming isolated pancreatic agenesis (Figure 1f). Abdominal ultrasound showed that the pancreatic head appeared echogenic and atrophied.

**Figure 2:**
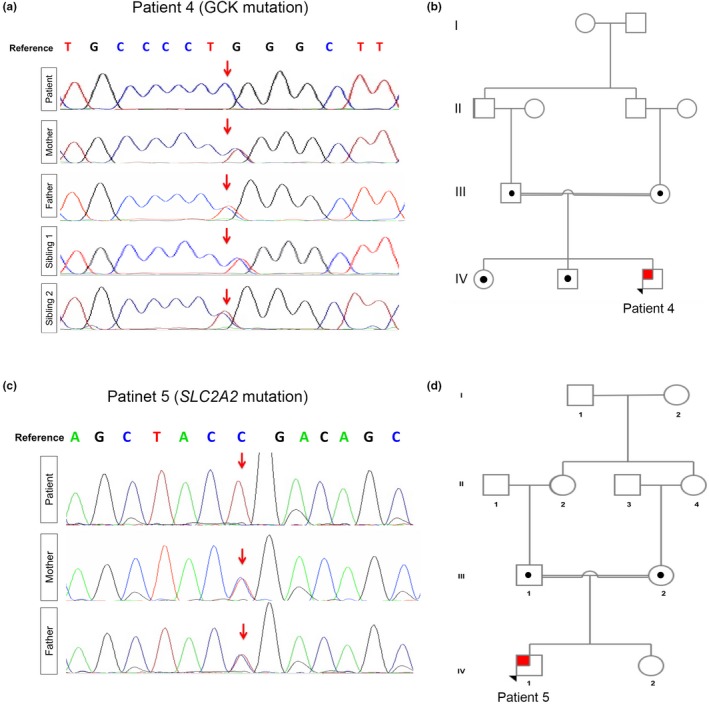
Patients with *GCK* and *SLC2A2* Recessive Mutations. **(**a) Chromatogram obtained using Sanger sequencing confirming *GCK* homozygosity (c.437T > C) in patient 4 and heterozygosity in both parents and siblings. (b) Pedigree chart significant for first‐degree consanguineous parents in family 4. (c) Chromatogram obtained using Sanger sequencing confirming *SLC2A2* homozygosity (c.901C > T) in patient 5 and heterozygosity in both parents. (d) Pedigree chart significant for first‐degree consanguineous parents in family 5. Squares indicate male persons, and circles female persons. The arrow indicates the proband. The open symbols, dotted symbols, and solid symbols represent nonvariant, heterozygote, and homozygous for mutations, respectively

Case No. 4 is an 11‐year‐old Egyptian boy, preterm born via NVD, to first‐degree consanguineous parents. The patient was diagnosed with PNDM when he wasone‐day‐old in the NICU. WES confirmed a homozygous c.437T > C missense mutation in Glucokinase (*GCK)* gene (NM_000162) resulting in p.Leu146Arg (Figure 2a). Sanger sequencing confirmed heterozygosity in both parents and siblings (Figure 2a,b).

Case No. 5 is a 24‐month‐old boy with FBS and severe proximal tubular dysfunction with failure to thrive and marked short stature. At 4 days of age, newborn screening revealed a mild increase in total galactose with normal galactose‐1‐phosphate uridylyl transferase (GALT) enzyme activity and all forms of Galactosaemia were excluded. At 18 days of life, the patient displayed characteristic biochemical findings of fasting hypoglycemia and postprandial hyperglycemia, a hallmark seen in FBS, with an elevated Hba1c level of 6.7% and low C‐peptide level diagnostic of PNDM. WES analysis was performed at two months of age detecting a homozygous nonsense c.901C > T mutation in exon 6 of *SLC2A2*(NM_000340), resulting in p.Arg301Ter (Figure 2c). Both parents were heterozygous carriers (Figure 2c). The *SLC2A2* mutation identified is expected to result in a truncated GLUT2 protein. The family pedigree indicates that our patient (individual IV‐1) has likely inherited an identical copy of the pathogenic *SLC2A2* allele through both parents due to common ancestors (greater grandparents‐ demonstrated in individuals I‐1 and/or 2 (Figure 2d).

**Figure 3:**
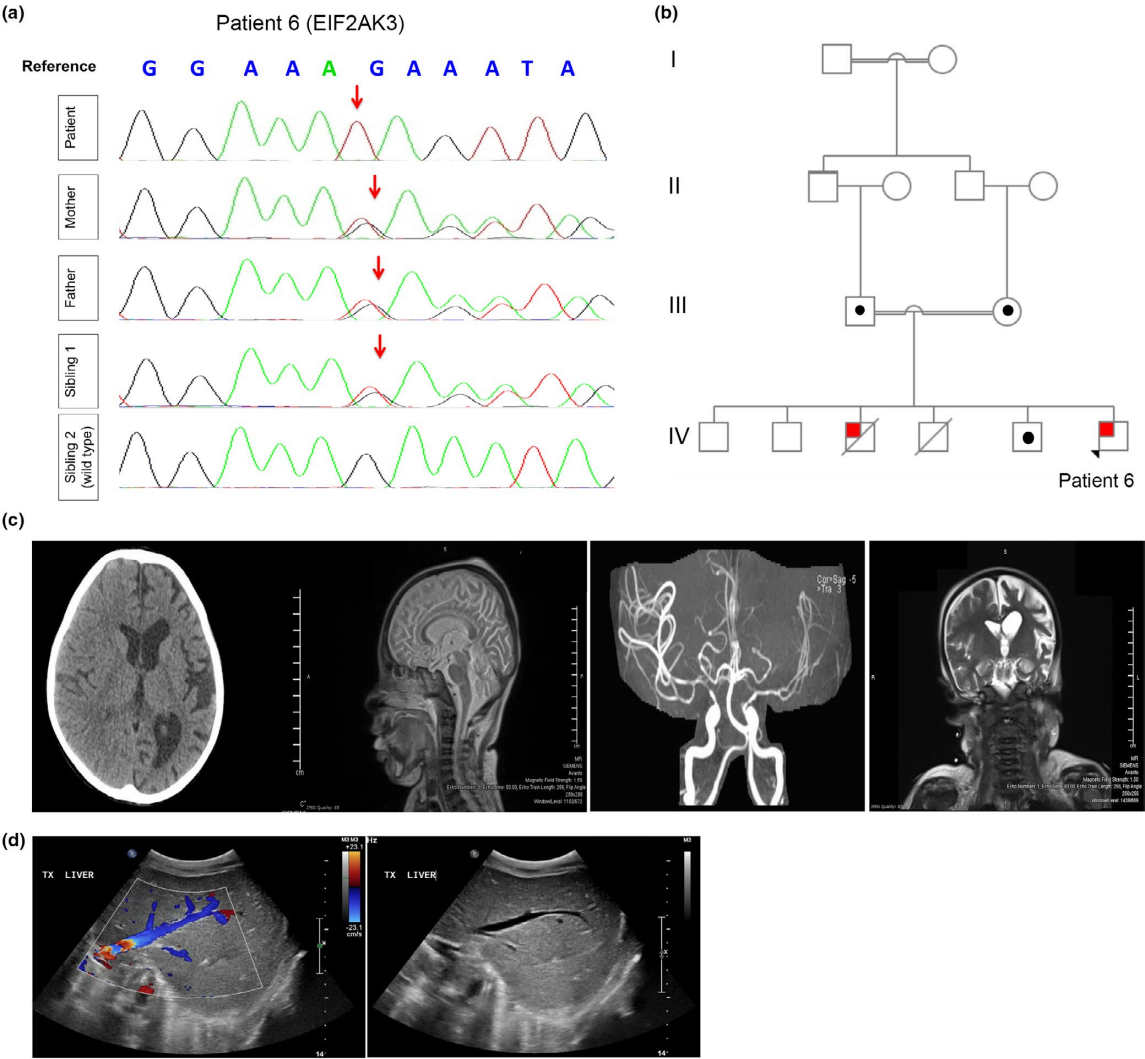
A Patient with Wolcott–Rallison Syndrome (WRS). (a) Chromatogram obtained using Sanger sequencing confirming homozygosity (c.1566_1569delGAAA in exon 9 of *EIF2AK3*) is patient 6 and heterozygosity in both parents and sibling 1. (b) Pedigree chart significant for first‐degree consanguineous parents in family 6. Squares indicate male persons, and circles female persons. The arrow indicates the proband. The open symbols, dotted symbols, and solid symbols represent nonvariant, heterozygote, and homozygous for mutations, respectively. (c) CT scans and MRA. CT of the head without contrast shows left cerebral atrophy with dilation of the left lateral ventricle. The MRA of the intracranial arteries showed a relative reduction in the caliber of the left MCA with a paucity of the distal branches of the let ACA and MCA compared to the right side. (d) US abdomen before liver transplant: Mild hepatomegaly. The liver is enlarged measuring 10.5 cm with coarse echotexture. US abdomen following liver transplant: The transplanted liver is normal in texture with no focal lesion. Normal flow signals are elicited. The hepatic veins and IVC are patent. Pancreatic head and body show normal thickness and echogenicity

Case No. 6 is an 8‐year‐old Qatari boy, born at full term, via NVD to a first‐degree consanguineous parent. Family history is significant for WRS in an older male sibling who passed away at the age of 12 due to liver failure. Targeted DNA analysis revealed a c.1570_1573delGAAA mutation in exon 9 of *EIF2AK3* (NM_004836). The four‐nucleotide deletion is predicted to cause a frameshift and results in aberrant mRNA processing (Brickwood et al., 2003). The patient is homozygous for this mutation. Analysis of DNA derived from the father and mother revealed a heterozygous *EIF2AK3* exon 9 deletion. *EIF2AK3* analysis was restricted to exon 9 and flanking sequences. One of the patient's siblings displayed similar heterozygosity of the same mutation (Figure 3a,b). The patient was diagnosed with PNDM at the age of 4 months and is maintained on insulin pump therapy. Mild cerebellar atrophy was noted as evident by magnetic resonance angiogram (MRA) (Figure 3c). The patient had a decompensated liver failure to which he had a successful timely liver transplantation. Abdominal US following liver transplant showed the transplant liver was normal in texture with no focal lesion (Figure 3d).

**Figure 4:**
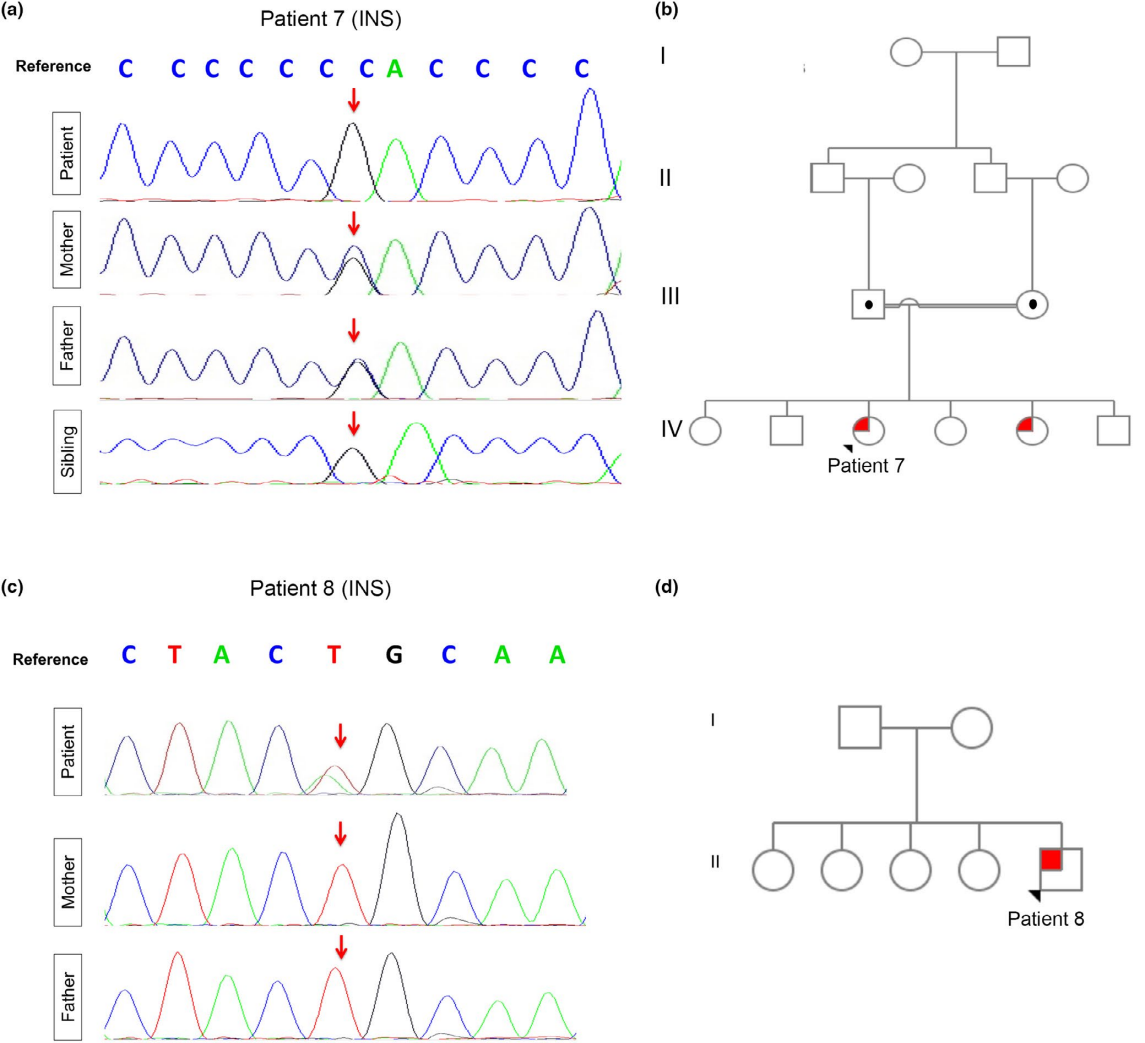
Patients with recessive and dominant novel *INS* mutations. (a) Chromatogram obtained using Sanger sequencing confirming INS homozygosity c.‐331C > G in patient 7 and her sibling. Heterozygosity shown in both parents. (b) Pedigree chart significant for first‐degree consanguineous parents in family 7. Squares indicate male persons, and circles female persons. The arrow indicates the proband. The open symbols, dotted symbols, and solid symbols represent nonvariant, heterozygote, and homozygous for mutations, respectively. (c) Chromatogram obtained using Sanger sequencing confirming *INS *de novo c.325T > A. Normal sequencing seen in both parents. (d) Pedigree chart Insignificant for consanguinity in family 7. Squares indicate male persons, and circles female persons. The arrow indicates the proband. The open symbols, and solid symbols represent nonvariant and heterozygote for the mutation, respectively

Recessive and dominant mutations in *INS* (NM_001185098) encoding insulin were detected in patients 7–8. Case No. 7 is an 11‐year‐old Qatari girl, born at full term via CS, to first‐degree consanguineous parents. The patient was diagnosed with PNDM before one month of age. WGS confirmed a homozygous mutation in *INS* c.‐331C > G (Figure 4a). Family history was significant for diabetes mellitus and atopic dermatitis in a younger sister, diagnosed at the age of two (Figure 4b). Sanger sequencing confirmed the mutation heterozygosity and homozygosity in the parents and an affected sister, respectively (Figure 4a). Case No. 8 is a 4‐year‐old Qatari boy born at full term via CS to nonconsanguineous parents who was diagnosed with PNDM at the age of two months presenting with diabetic ketoacidosis (DKA). Genome sequencing revealed a novel de novo c.325T > A variant in *INS* (p.Cys109Ser) predicted to be deleterious based on PolyPhen‐2 (Polymorphism phenotyping v2) score (Figure 4c,d).

**Figure 5:**
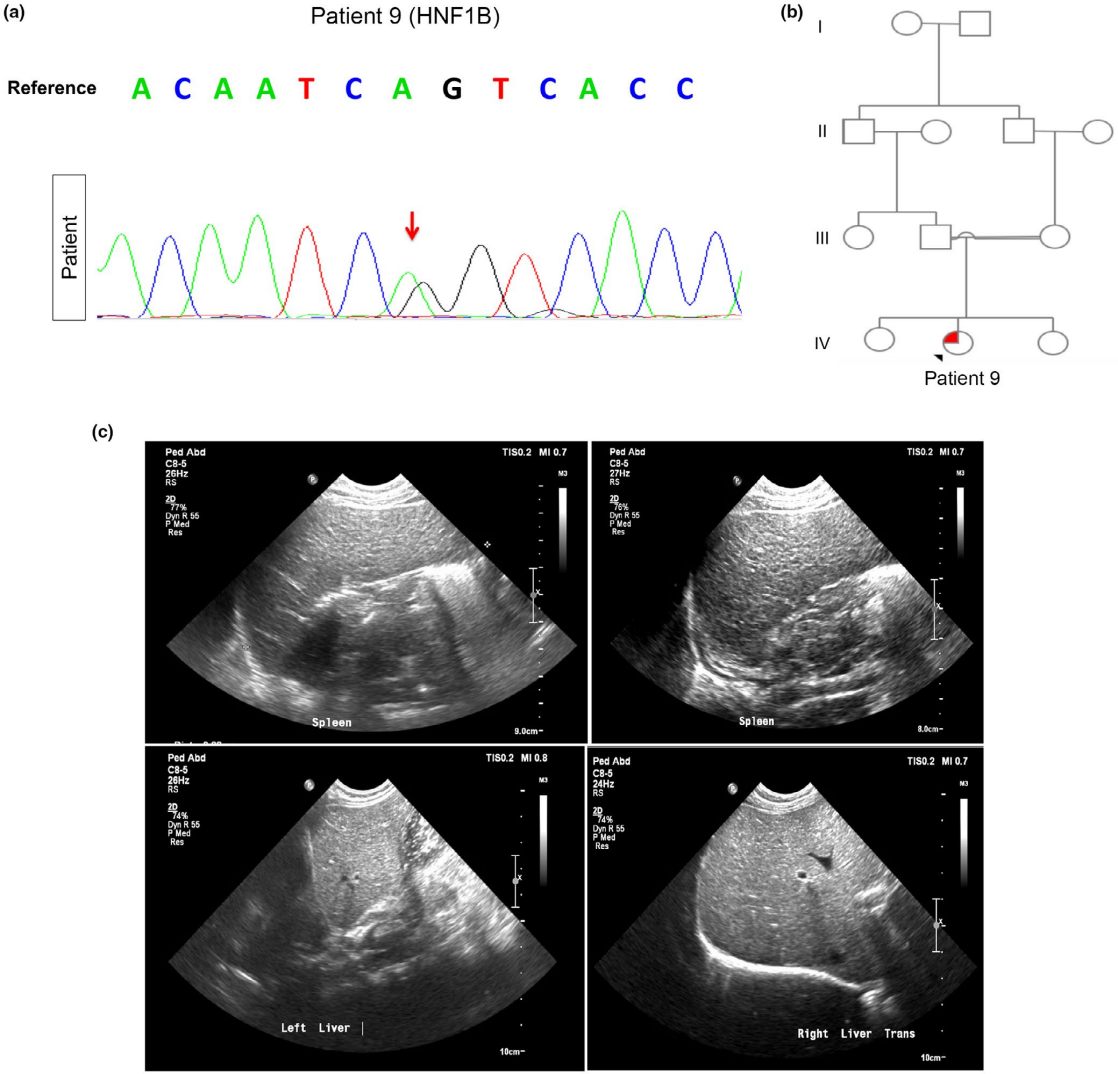
PNDM due to Novel de novo* HNF1B* Heterozygous Mutation. (a) Chromatogram obtained using Sanger sequencing confirming *HNF1B* heterozygosity (c.1099A > G, p.Ser367Gly) in patient 9. (b) Pedigree chart significant for first‐degree consanguineous parents in family 9. Squares indicate male persons, and circles female persons. The arrow indicates the proband. The open symbols, and solid symbols represent nonvariant and heterozygote for the mutation, respectively. (c) Abdominal ultrasound showing hepatosplenomegaly

Case No. 9 is a 4‐year‐old female of Afghani origin, born to first‐degree consanguineous parents. The patient was diagnosed with PNDM, failure to thrive, and congenital hypothyroidism at the age of three days old. Genome analysis revealed a novel heterozygous c.1099A > G mutation in *HNF1B* (NM_000458) resulting in p.Ser367Gly (Figure 5a,b). The patient also has hypoalbuminemia, anemia of chronic disease, fungal cellulitis, bilateral hydronephrosis, and mild hepatosplenomegaly (Figure 5c). The patient further displayed severe electrolyte imbalance attributed to her abnormal renal status (phosphate 1.32, reference 1.45–2.33 mmol/L; magnesium 0.79, reference 0.87–1.19 mmol/L; sodium 130, reference 135–145 mmol/L, high potassium 5.8, reference 3.5–5.2 mmol/L) and low albumin of 24 g/L; reference 35–46).

## 4 DISCUSSION

In this study, we discuss the first data on PNDM from The State of Qatar. In our cohort, 100% of patients had an identifiable genetic etiology for their neonatal diabetes. Recessive *PTF1A* distal enhancer deletions mutations were the most frequent cause of PNDM in the state in Qatar, followed by *INS* gene mutations. The incidence of PNDM in the State of Qatar during the period 2001–2016 is 1:22,938 live births among the indigenous Qatari population; at least 10 times worldwide estimates (Slingerland et al., 2009). Our calculated incidence is similar to the highest PNDM incidence reported from Northwest Saudi Arabia, reaching 1:21,196 (Habeb et al., 2012). Other closely related Arabian Gulf regions illustrated similar incidence of PNDM; 1:45,787 in Oman (Bappal, Raghupathy, Silva, & Khusaiby, 1999) and 1:31,900 live births in the United Arab Emirates (UAE) (Deeb et al., 2016). We anticipate that the principal cause for the high incidence of PNDM and other rare autosomal recessive disorders is attributed to a combination of consanguinity and low birth rates in Qatar. Specifically, in the Bedouin/Arab Qataris (Q1) subpopulation, the practice of within‐tribal marriage continues to cause an increased level of homozygosity compared with other populations (Hunter‐Zinck et al., 2010; Rodriguez‐Flores et al., 2016).

### 4.1 *PTF1A* enhancer deletions in NDM

Our data analysis shows that *PTF1A* enhancer deletions (10:23502416–23510031) are the most common cause of PNDM in the state of Qatar with only a few previous cases reported in the literature (Weedon et al., 2014). Mutations in other transcription factors, such as *GATA6*, *PDX1*, and *GATA4* can also lead to pancreatic agenesis (De Franco et al., 2013; Houghton et al., 2016; Shaw‐Smith et al., 2014; Stoffers, Zinkin, Stanojevic, Clarke, & Habener, 1997). Recessive coding mutations in *PTF1A* cause pancreatic agenesis with cerebellar agenesis and neurological sequel (Al‐Shammari, Al‐Husain, Al‐Kharfy, & Alkuraya, 2011; Sellick et al., 2004; Tutak et al., 2009). Using linkage, whole genome sequencing, and epigenomic annotation in human embryonic stem cell (hESC)‐derived pancreatic progenitor cells, autosomal recessive defects in the noncoding region of *PTF1A* underlying isolated pancreatic agenesis have been revealed (Weedon et al., 2014). A recent study reported patients with isolated pancreatic agenesis due to compound heterozygous truncating *PTF1A* mutations (Gabbay, Ellard, Franco, & Moises, 2017). In the current study, whole genome sequencing on three patients with isolated pancreatic agenesis descending from two unrelated Arabian Peninsula consanguineous families, revealed a previously reported distal *PTF1A* enhancer recessive mutation (chr10:23502416–23510031) that included the entire putative enhancer (Weedon et al., 2014). The mutation locates in a short (~400bp) evolutionary conserved region and is predicted to prevent the enhancer activity by preventing transcription factors *FOXA2* and *PDX1* from binding (Weedon et al., 2014). Taken together, these findings indicate that the site of *PTF1A* mutations (coding or noncoding mutations), determines the phenotypic characteristics of the patients. Furthermore, it suggests an important role of the noncoding sequences during pancreatic endocrine and exocrine development.

### 4.2 Glucokinase (GCK) gene mutations in PNDM

Homozygous inactivating mutations (loss of function) in *GCK* also contribute to the development of PNDM (Gloyn, 2003; Njolstad et al., 2003, 2001). Our patients previously reported that inactivating *GCK* mutation (Raimondo et al., 2014) is expected to prevent the increase in the ATP/ADP ratio needed for the closure of the K_ATP_ channels, which consequently prevents the opening of the voltage‐gated calcium channels, ultimately resulting in the failure to initiate insulin release (Koster, Marshall, Ensor, Corbett, & Nichols, 2000; Njolstad et al., 2003). Interestingly, mutations in *PDX1/IPF‐1* and *GCK* cause MODY in the heterozygous state, while homozygous mutations in these genes cause PNDM (Osbak et al., 2009; Stoffers, Ferrer, Clarke, & Habener, 1997).

### 4.3 PNDM in Fanconi–Bickel Syndrome (FBS)

The functional loss of GLUT2 is compatible with the biochemical findings observed in patients with FBS (Santer et al., 1997). Our patient's homozygous nonsense c.901C > T mutation in *SLC2A2* has been originally described by Fanconi and Bickel in 1949 and later by Santer et al. in 1997 (Santer et al., 1997). The data on insulin levels in FBS are limited; however, our patient demonstrates decreased C‐peptide level and insulin level, and a high HbA1c. PNDM in association with FBS have been described before in regions with high consanguinity (Habeb et al., 2012). A study on homozygous mice deficient in GLUT‐2 showed that they are relatively hypo‐insulinemic (Guillam et al., 1997). Indeed, studies have shown that GLUT2 is not the main glucose transporter in human beta cells and that *SLC2A2 *mutations drive the glucose dysregulation observed in carriers of *SLC2A2 *variants (McCulloch et al., 2011). An alternative contribution of GLUT2 could be via its effect on beta‐cell proliferation as *Nkx6.1* controls this process by regulating GLUT2 expression (Taylor, Liu, & Sander, 2013). GLUT2 reconstitution in a KO model of *Nkx6.1* increased *Ccnd2* expression and the number of insulin positive cells, whereas expression of other *Nkx6*.1‐regulated genes remained significantly reduced (Taylor et al., 2013). These findings indicate that GLUT2 reexpression rescues beta‐cell proliferation.

### 4.4 PNDM in Wolcott–Rallison Syndrome (WRS)

WRS is caused by inactivating mutation in the eukaryotic translation initiation factor 2‐alpha kinase 3 (*EIF2AK3)* gene, also called protein kinase R (PKR)‐like endoplasmic reticulum kinase (PERK), a highly expressed protein in pancreatic islets (Shi et al., 1999). Clinical phenotypes of WRS include PNDM, abnormal bone histology (epiphyseal and spondyloepiphyseal dysplasia), cerebellar cortical dysplasia, hepatic and renal dysfunction, cardiomegaly, and mental retardation (Stewart et al., 1996; Thornton, Carson, & Stewart, 1997). PERK plays an important role in recognizing unfolded proteins in the ER and activating the transcription of several genes to increase the ER‐folding capacity to initiate apoptosis (Brostrom & Brostrom, 1998). Our patient displayed the characteristic findings of WRS with a previously reported *EIF2AK3* mutation (Brickwood et al., 2003). The patient had a decompensated liver failure in which he had successful timely liver transplantation, being among the fewest reported worldwide (Rivera et al., 2016; Tzakis et al., 2015). The promising outcomes of our first reported patient to undergo liver transplant from Qatar raises the chances for an improved life expectancy for WRS patients, especially with the success of the combined liver, pancreas, and kidney transplant in children with WRS (Habeb et al., 2015; Tzakis et al., 2015). Proper genetic counseling was provided to the only Qatari family with WRS, advising preimplantation genetic diagnosis (PGD) for future pregnancies.

### 4.5 *INS* gene mutations in PNDM

The mutations identified in this study illustrate several key mechanisms causing aberrant insulin biosynthesis. Recessive and novel dominant mutations in *INS* were found in our cohort. *INS* gene mutations have been associated with PNDM (Edghill et al., 2008; Garin et al., 2010; Stoy et al., 2007). Hyperglycemia occurs due to decreased insulin biosynthesis, in which most of the reported missense heterozygous mutations are expected to cause an abnormal proinsulin folding. An accumulation of the misfolded protein in the endoplasmic reticulum (ER) consequently occurs, resulting in ER stress and beta‐cell apoptosis (Liu, Hodish, Rhodes, & Arvan, 2007). Our identified de novo novel variant in *INS* is expected to result in aberrant proinsulin trafficking and consequent ER stress and beta‐cell apoptosis. The recessive homozygous point mutation is expected to impair the activity of the INS promoter (Niu et al., 2007). The recessive INS c.‐331 C > G sequence, located between the E1 and A1 elements, is conserved among a subset of mammalian species (Niu et al., 2007). The previously reported CC dinucleotide that is mutated in our patient creates part of a critical positive cis regulatory sequence of the INS promoter (Garin et al., 2010). Formation of the canonical CCACC binding‐site motif for Kruppel‐like zinc‐finger proteins is dependent on the cis regulatory sequence, in which GLIS3 (zinc‐finger transcription factor) exhibits sequence‐specific binding to this region (Senee et al., 2006). Most of the reported heterozygous missense mutations in the coding region of INS are expected to disrupt the folding of the proinsulin molecule (Polak et al., 2008), resulting in misfolded protein accumulation in the ER, and consequently inducing ER stress and beta‐cell apoptosis (Herbach et al., 2007; Liu et al., 2007).

### 4.6 *HNF1B* gene mutations in PNDM

*HNF1B* is a member of the POU‐homeobox family of basic helix‐loop‐helix proteins that bind to DNA as dimers (Aguilar‐Bryan & Bryan, 2008). *HNF1B* is crucial for the differentiation of visceral endoderm (Barbacci et al., 2004; Bellanne‐Chantelot et al., 2004; Lu, Rha, & Chi, 2007). Several reports of patients with *HNF1B* mutation (maturity‐onset diabetes of the young; MODY 5) display pancreatic atrophy, a phenomenon that could be attributed in part to *HNF1B* being a key member of the transcriptional factors network that controls the differentiation of the endodermal pancreatic precursor cells (Poll et al., 2006; Wang et al., 2004). *HNF1B* tissue distribution includes the pancreas, liver, thymus, gut, kidney, genital tract, and lung (Hiesberger et al., 2004; Ma et al., 2007; Okita et al., 1999; Tanaka et al., 2004). Mutations in *HNF1B* have been associated with TNDM (Yorifuji et al., 2004). The clinical characteristics in those neonatal diabetic patients with *HNF1B* mutations include hyperglycemia, renal abnormalities and genital malformations (e.g., vaginal and Mullerian aplasia) (Iwasaki et al., 1998; Lindner et al., 1999; Yorifuji et al., 2004). Our patient's novel variant in *HNF1B* reflects a potentially pathogenic variant, however, further functional validation is ongoing to evaluate its effect in early pancreatic development. PNDM caused by mutation of the pseudo‐POU domain of *HNF1B* due to germline mosaicism has been reported (Edghill et al., 2006; Yorifuji et al., 2004). Heterozygous *HNF1B* mutations have been first described in 2004 in two siblings with TNDM, and later in another patient diagnosed with PNDM, yet all presenting with variable phenotypes (pancreatic atrophy, polycystic kidney, and exocrine insufficiency (Edghill et al., 2006; Yorifuji et al., 2004). It remains unclear why neonatal diabetic patients with the same *HNF1B* mutation present with discordant phenotypes (dysplastic kidneys), suggesting that additional factors (modifier genes and environmental factors) can have a significant influence on the phenotypic expression of *HNF1B* mutations (Yorifuji et al., 2004). We report a completely novel mutation in *HNF1B* with a complex phenotype consistent with the gene's function in early visceral endoderm (Cereghini, 1996). It is possible that our patient's mutation is subjected to nonsense mediated mRNA decay (NMD), where haploinsufficiency is a predicted in vivo mechanism responsible for the phenotypes associated with heterozygous nonsense alleles (Inoue et al., 2004). However, *HNF1B* heterozygous mutations phenotypes could either develop from defective DNA binding and/or diminished transactivation capacity through compromised recruitment of coactivator proteins. *HNF1A* and *HNF1B* specifically mediate transcriptional activation by exhibiting coregulators and corepressors pattern of interaction, functioning via chromatin remodeling (Barbacci et al., 2004).

### 4.7 Building a comprehensive mutation repository of PNDM in The state of Qatar

We have successfully described the clinical and genetic characteristics in our PNDM cohort, with a similar incidence described in other highly consanguineous Arabian Peninsula populations (Demirbilek et al., 2015; Habeb et al., 2012). Identifying the genetic etiology in our entire PNDM cohort was achievable using whole genome analysis following failure of previous limited WES to reveal the causal alleles. The identification of homozygous *INS* mutation in patient 7 refined the diagnosis of diabetes in her siblings, who was originally diagnosed with type 1 DM (T1DM) at first presentation. The variation in disease onset illustrates possible benefit from next‐generation sequencing in screening other family members who could develop DM. Our data reflect easier recent access to genome analysis in the State of Qatar. Although targeted gene panel reduces incidental findings and allows for higher sequencing depth compared to WES and WGS for the targeted genes at a low cost, it does not allow for the identification of novel genes (Stenson et al., 2014). Furthermore, the larger the gene panel, the less cost effective it becomes compared to WES. On the other hand, the targeted WES covers the most studied part of the genome at a lower cost compared to WGS, however, introns and regulatory domains are usually missed (Bamshad et al., 2011). WGS allows for the identification of intergenic regions and exerts a superior role for copy number variation (CNV) detection (Belkadi et al., 2015). Although WGS cons are exemplified by the increased calls and analysis complexity, the value of utilizing WGS versus targeted sequencing in our cohort allows for the discovery of novel mutations in regulatory noncoding regions (Goldstein et al., 2013; Sims, Sudbery, Ilott, Heger, & Ponting, 2014). Use of this reference in Qatar will lead to higher quality interpretation for both individual genomes and Mendelian disease studies, an important milestone which will continues to provide new perspectives into monogenic disorders in highly consanguineous populations like in Qatar. Finally, the role of noncoding variants to PNDM remains largely unknown, with emerging evidence of monogenic diseases causing mutations in distal regulatory elements (Cooper et al., 2010; Sankaran et al., 2011; Smemo et al., 2012; Spielmann et al., 2012). Although whole genome sequencing could potentially identify causal noncoding variants, distinguishing functional noncoding causal variants from millions of noncoding variants remain a challenge. However, our data provide promise that identification of novel regulatory element mutations continue to provide new perspectives into PNDM causal alleles.

In conclusion, identifying the most common cause of PNDM in our Qatari cohort helps in refining future diagnostic approaches toward improving genetic diagnosis in the field of NDM. Our comprehensive genetic analysis suggests an important role of the noncoding sequences during pancreatic endocrine and exocrine development via identifying the distal *PTF1A* enhancer region. This study further establishes an improved bioinformatics approach tackling PNDM genome analysis studies in closely related Arabian and MENA populations. Enhanced read depth and variant sensitivity further demonstrates the utility of our PNDM reference genome. Use of this reference in the native Arab population of Qatar of Arabian Peninsula origins will continue to help in achieving an enhanced analysis for both individual genomes and monogenic diabetes disorders.

## 5 ETHICS STATEMENT

Written informed consent was obtained from the parents of the patient for the participation in the study and the publication of this case report and accompanying images. A copy of the written consents from both parents is available for review by the editor of this journal.

## CONFLICT OF INTEREST

The authors declare the absence of any commercial or financial relationships that could be construed as a potential conflict of interest.
