# Supplementary figures and images for: The clinical and genetic characteristics of permanent neonatal diabetes (PNDM) in the state of Qatar
Source: Mol Genet Genomic Med. 2019 Aug 23;7(10):e00753. doi: 10.1002/mgg3.753 (PMC6785445; doi:10.1002/mgg3.753)

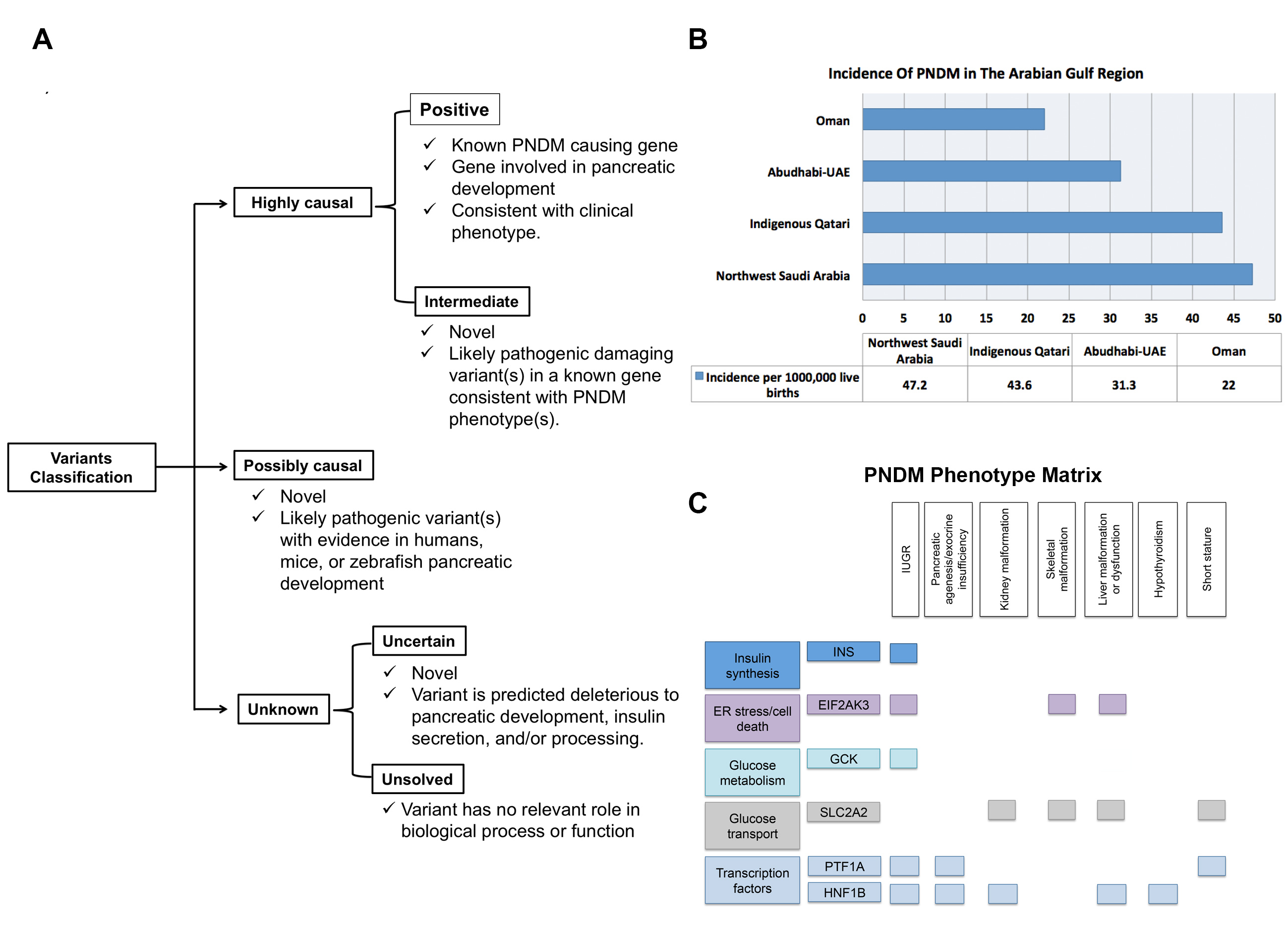

Supplement: Supplementary file 1 [file MGG3-7-e00753-s001.tif]
