# Supplementary material for: The clinical and genetic characteristics of permanent neonatal diabetes (PNDM) in the state of Qatar
Source: Mol Genet Genomic Med. 2019 Aug 23;7(10):e00753. doi: 10.1002/mgg3.753 (PMC6785445; doi:10.1002/mgg3.753)
Supplement: Supplementary file 2 [file MGG3-7-e00753-s002.docx]

Table 1 Primers used for Sanger Sequencing

| **Gene** | **Sequence** | **Primer design** |
| --- | --- | --- |
| SLC2A2 | c.901C>T | F: TGGCCTGAGTTGTTTCAACC  R: TTCCGGAAAATTGCTGAGCC |
| EIF2AK3 | c.1566_1569delGAAA | F: GGGAGAGGAACAAACGAAGC  R: CTTTGGTGGAGCAGTAGGGA |
| INS | c.-331C>G | F: GAGGAAGAGGTGCTGACGAC  R: CTTCTGATGCAGCCTGTCCT |
| INS | c.325T>A | F: CCCTGACTGTGTCCTCCTGT  R: GGCTGGTTCAAGGGCTTTAT |
| GCK | c.437T>C | F: GCATCTTCCAGCTCTTCGAC  R: GGGCTACATTTGAAGGCAGA |
| HNF1B | c.1099 A>G | F: CCCTGGTGGCACTAATGTTC  R: ATCAGCTCCAGAGCGACAAT |
