# Supplementary material for: The clinical and genetic characteristics of permanent neonatal diabetes (PNDM) in the state of Qatar
Source: Mol Genet Genomic Med. 2019 Aug 23;7(10):e00753. doi: 10.1002/mgg3.753 (PMC6785445; doi:10.1002/mgg3.753)
Supplement: Supplementary file 3 [file MGG3-7-e00753-s003.docx]

**Supplementary Table 2. The incidence of PNDM during the period 2001-2016 in the Qatari population**

| **Year** | **Live Births - indigenous Qatari** | **Live Births - Non- indigenous Qataris** | **Live Births - Total** |
| --- | --- | --- | --- |
| **2001** | **5855** | **6263** | **12118** |
| **2002** | **5924** | **6276** | **12200** |
| **2003** | **6193** | **6663** | **12856** |
| **2004** | **6488** | **6702** | **13190** |
| **2005** | **6260** | **7141** | **13401** |
| **2006** | **6563** | **7557** | **14120** |
| **2007** | **7178** | **8503** | **15681** |
| **2008** | **7621** | **9993** | **17614** |
| **2009** | **7532** | **11055** | **18587** |
| **2010** | **7757** | **11777** | **19534** |
| **2011** | **7732** | **13070** | **20802** |
| **2012** | **7228** | **14541** | **21769** |
| **2013** | **8030** | **16001** | **24031** |
| **2014** | **8032** | **17575** | **25607** |
| **2015** | **8292** | **18434** | **26726** |
| **2016** | **8005** | **18918** | **26923** |
|  |  |  |  |
| **Total Live Births** | **114690** | **180469** | **295159** |
| **Number of Neonatal DM** | **5** | **4** | **9** |
| **Incidence (per live births)** | **1: 22,938** | **1:45,117** | **1:32,795** |
|  |  |  |  |
| **Incidence per 1,000,000 births** | **43.6** | **22.2** | **30.5** |
| **95% CI per 1,000,000 births** | **14.2-101.7** | **6.0-56.7** | **13.9-57.9** |
